# Proprotein Convertase Subtilisin Kexin Type 9 (PCSK9) Beyond Lipids: The Role in Oxidative Stress and Thrombosis

**DOI:** 10.3390/antiox11030569

**Published:** 2022-03-16

**Authors:** Vittoria Cammisotto, Francesco Baratta, Paola G. Simeone, Cristina Barale, Enrico Lupia, Gioacchino Galardo, Francesca Santilli, Isabella Russo, Pasquale Pignatelli

**Affiliations:** 1 Department of Clinical Internal, Anesthesiological and Cardiovascular Sciences, Sapienza University of Rome, 00161 Rome, Italy; vittoria.cammisotto@uniroma1.it (V.C.); francesco.baratta@uniroma1.it (F.B.); 2 Department of General Surgery and Surgical Speciality Paride Stefanini, Sapienza University of Rome, 00161 Rome, Italy; g.galardo@policlinicoumberto1.it; 3 Department of Medicine and Aging, and Center for Advanced Studies and Technology (CAST), “G. D’Annunzio” University Foundation, 66100 Chieti, Italy; paolagsimeone@gmail.com (P.G.S.); francesca.santilli@unich.it (F.S.); 4 Department of Clinical and Biological Sciences, University of Turin, 10043 Turin, Italy; cristina.barale@unito.it (C.B.); isabella.russo@unito.it (I.R.); 5 Department of Medical Sciences, University of Turin, 10126 Turin, Italy; enrico.lupia@unito.it; 6 Mediterranea Cardiocentro, 80133 Napoli, Italy

**Keywords:** proprotein convertase subtilisin/kexin type 9 (PCSK9), platelets, thrombosis, anti-PCSK9, oxidative stress

## Abstract

Proprotein convertase subtilisin/kexin type 9 (PCSK9), mainly secreted in the liver, is a key regulator of cholesterol homeostasis inducing LDL receptors’ degradation. Beyond lipid metabolism, PCSK9 is involved in the development of atherosclerosis, promoting plaque formation in mice and human, impairing the integrity of endothelial monolayer and promoting the events that induce atherosclerosis disease progression. In addition, the PCSK9 ancillary role in the atherothrombosis process is widely debated. Indeed, recent evidence showed a regulatory effect of PCSK9 on redox system and platelet activation. In particular, the role of PCSK9 in the activation of nicotinamide adenine dinucleotide phosphate (NADPH) oxidase (Nox2) system, of MAP-kinase cascades and of CD36 and LOX-1 downstream pathways, suggests that PCSK9 may be a significant cofactor in atherothrombosis development. This evidence suggests that the serum levels of PCSK9 could represent a new biomarker for the occurrence of cardiovascular events. Finally, other evidence showed that PCSK9 inhibitors, a novel pharmacological tool introduced in clinical practice in recent years, counteracted these phenomena. In this review, we summarize the evidence concerning the role of PCSK9 in promoting oxidative-stress-related atherothrombotic process.

## 1. Introduction

The World Health Organization (WHO) estimated that around one third of global deaths are due to cardiovascular disease (CVDs); hence, both primary and secondary CVD prevention is one of the most important challenges of our times.

A fundamental aspect of CVD development is represented by atherosclerosis, the anatomopathological process of large arteries leading to coronary artery disease (CAD), ischemic stroke and other CVDs. Low-density lipoprotein cholesterol (LDL-c) is the only risk factor meeting the causality criteria for atherosclerosis [1]. However, the inflammatory process plays a key role in the promotion of LDL-c transcytosis across the endothelium and LDL-c retention in the artery wall [2]. In the arterial intima, LDL-c itself exerts a prooxidative and proinflammatory role [2]. In this site, LDL-c particles undergo enzymatic and non-enzymatic oxidative processes, inducing oxidized-LDL (ox-LDL) [3,4].

Notably, platelets not only play a role in thrombus growth but, upon stimulation, induce LDL oxidation, contributing to the atherosclerotic process. Indeed, platelets, activated by inflammatory stimulus, adhere to endothelium cells and amplify immuno-inflammatory process, favoring innate immunity cells’ chemotaxis [5] and the release of pro-oxidative and pro-inflammatory mediators [6], leading to the production of prothrombotic agents such as ox-LDL [7].

The interplay among lipid, platelets and inflammatory stimuli exemplifies the complexity of CVD prevention, which must be based on a multifactorial approach built on lipid-lowering strategies and the management of residual cardiovascular risk, which is beyond LDL-c control [8].

Proprotein Convertase Subtilisin Kexin Type 9 (PCSK9) is an enzyme of the serine proteases classes first described in 2003 [9]. This enzyme is synthesized in the cell as a soluble zymogen and converted into its active form after an autocatalytic process in the endoplasmic reticulum [10].

PCSK9 is a critical regulator of cholesterol homeostasis, acting as an inhibitor of the LDL receptors (LDLR) pathway [11]. Mostly secreted in the liver, PCSK9 is also expressed in the arterial wall, where it can influence local hemostasis and atherosclerosis [12].

Given the strong correlation between dyslipidemia and CVD, LDL-C reduction by inhibiting the LDLR-PCSK9 axis drastically contributes to reduced CVD risk [13,14].

In the last decade, two different human monoclonal antibodies were developed, namely alirocumab and evolocumab. PCSK9 inhibitors (PCSK9-i) bind circulating PCSK9, and prevent PCSK9-LDL-LDLr complex production and the subsequent LDL turnover [15]. Their use reduces circulating LDL-c by about 40–65% when used on top of statins [16,17].

Phase-3 interventional studies demonstrated that both alirocumab and evolocumab reduce cardiovascular events beyond the lipid-lowering effect of the PCSK9 inhibitors (PCSK9i) [18,19], inducing investigations into the so-called pleiotropic effects of inhibiting PCSK9.

In recent years, a growing number of studies were conducted to explore the ancillary effects of PCSK9-i, which may explain the cardiovascular protective effect of these drugs.

The aim of this narrative review is to discuss the latest evidence on the role of PCSK9 in the atherothrombotic process, via oxidative stress and platelet activity modulation. MEDLINE research was conducted on works published until 15 January 2022, combining “PCSK9” and “proprotein convertase subtilisin kexin type 9” with “oxidative stress”, “anti-oxidant”, “platelet”, “thrombosis”, “atherothrombosis”, “anti-thrombotic”. 

## 2. Oxidative Stress

### 2.1. Role of Oxidative Stress in the Atherothrombotic Process

Numerous evidence suggests that oxidative stress is a relevant actor involved in endothelial dysfunction and, consequently, in the biological mechanism, including platelet activation, at the basis of CVD progression [20]. Oxidative stress is a phenomenon caused by the imbalance between the production of oxygen reactive species (ROS) and the biological systems’ ability to detoxify these reactive products [21]. Several enzymatic systems seem to be involved in this process, such as xanthine oxidase, the nicotinamide adenine dinucleotide phosphate (NADPH) oxidases, mitochondrial sources, and nitric oxide synthase (NOS) [22]. Among them, NADPH oxidase plays a main role, as shown by the almost complete cellular ROS suppression in patients with a hereditary deficiency of Nox2, the catalytic subunit of the enzyme [23]. Finally, antioxidant molecules play a key role in maintaining the physiological redox balance. Along with albumin, uric acid represents the main circulating non-enzymatic antioxidant system in human [24,25]. Other non-enzymatic antioxidants include Vitamins E and C, Coenzyme Q10 and polyphenols that are integrated through the diet [26] and glutathione (GSH), the most abundant low-molecular-weight thiol compound synthesized in cells [27].

Vascular inflammation and oxidative stress play an important role in the pathogenesis of CVDs, from the initiation of atherosclerosis through to the progression of plaques until thrombotic complications [28,29]. Platelets, which play a key role in the atherothrombosis mechanism via the release of inflammatory and pro-thrombotic molecules, are also able to produce ROS [30]. Currently, we know that, upon activation, platelet ROS, mainly produced by NADPH oxidase, are implicated in several processes, including the propagation of platelet activation, releasing platelet agonists such as Adenosine Diphosphate (ADP), forming isoprostanes and ox-LDL, and releasing pro-atherogenic molecules such as CD40 ligand (CD40L) [31]. Several studies demonstrated a key role for Nox2-mediated ROS formation in eliciting platelet activation. In particular, the interplay between Nox2, a NADPH oxidase isoform and platelet activation was first studied in platelets derived from X-linked chronic granulomatous disease (X-CGD) patients [32]. These patients, characterized by a genetic deficiency of NADPH oxidase [33], showed a reduction in CD40L expression and production upon collagen- and thrombin-stimulation, in addition to the almost complete suppression of superoxide anion (O^2−^) radical production [32].

Lipid peroxidation by ROS derived from NADPH oxidase activity results in initiation of the inflammatory signaling, an early event in atherosclerotic plaque development [34]. Based on this evidence, platelets represent a source of ROS that contributes to LDL oxidation, and, in turn, determines enhanced LDL uptake by macrophages via an oxidative stress-mediated mechanism. Two platelet receptors that are important in the development of atherosclerosis are scavenger receptors, such as the cluster of differentiation 36 (CD36) and lectin-like oxidized low-density lipoprotein receptor 1 (LOX-1). CD36 is a ligand for ox-LDL, and previous studies have shown that their interaction triggers signaling pathways, activating platelets, and inducing the expression of P-selectin and the activation of integrin αIIbβ3 (a fibrinogen receptor) [35]. Furthermore, the binding of ox-LDL to CD36 was associated with platelet hyperreactivity and plays a crucial role in the prothrombotic phenotype [35] via many mechanisms including NADPH oxidase activation and ROS production.

ROS production by ox-LDL/CD36 requires Src-family kinases, PKC-dependent phosphorylation and the activation of Nox2, and is inhibited in vitro by CD36 inhibitors and Nox2 inhibitor (gp91ds-tat) [36]. Similarly, ROS production is inhibited in Nox2^(−/−)^ mice [36], confirming the crucial role of Nox2. This evidence supports the hypothesis that platelet activation via specific ox-LDL/CD36 is mediated by Nox2 [37].

As a consequence, a vicious circle of LDL oxidation and platelet activation occurs [38]. LOX1 is also involved in the regulation of ox-LDL uptake by both endothelial cells and platelets. LOX1 expression is atheroma-related and not native and, like CD36, its interaction with ox-LDL induces platelet activation and aggregation, contributing to thrombus formation [39].

### 2.2. PCSK9 and Oxidative Stress

Recent data highlighted that PCSK9 has pro-atherogenic functions independently of its regulatory effect on plasma lipid levels. PCSK9 is highly expressed in vascular smooth muscle cells and in human atherosclerotic plaques, and its expression is regulated by its many pro-atherogenic mediators, including the production of NADPH oxidase-derived ROS and, in turn, ox-LDL formation [40]. In this regard, scavenger receptors such as CD36 and LOX-1 promote the endocytosis of ox-LDL particles in inflammatory cells and their expression is increased by inflammatory stimulus such as lipopolysaccharide (LPS) or tumor necrosis factor-α (TNFα) [41]. Once internalized, ox-LDL can induce the overexpression of PCSK9, perpetrating a proatherogenic stimulus, as PCSK9, in turn, are able to stimulate Nox2-mediated ox-LDL formation [36].

The relationship between PCSK9, NOX and the atherogenic ox-LDL is described in both animal models and humans at high risk for CVE. In particular, the interplay between PCSK9 and ROS production was studied in p47phox and gp91phox knockout mice models [41,42]. The authors found a relationship between PCSK9 and ROS production by studying endothelial cells (ECs) and smooth muscle cells (SMCs) treated with different concentrations of recombinant human PCSK9 protein (hPCSK9). The data showed that hPCSK9-treated ECs and SMCs produce more ROS in a concentration-dependent manner [42]. Furthermore, a cross-sectional study proved the correlation between plasma PCSK9 levels and soluble markers of endothelial damage (sICAM-1 and sVCAM-1) in patients with chronic kidney disease (CKD) [43].

Moreover, PCSK9 administration influenced NADPH oxidase activation, enhancing the expression of NADPH oxidases subunits such asp47 and gp91 phox [42]. Ding et al. demonstrated that macrophages from gp91phox^−/−^, p47phox^−/−^, and p22phox^−/−^ mice showed lower levels of ROS generation compared to wild-type (WT) mice. Furthermore, PCSK9 gene overexpression induced by plasmid transfection enhances ROS production in macrophages from knockout mice [41].

Several clinical studies showed that PCSK9 is involved in atherosclerotic inflammation. In fact, there is an association between serum PCSK9 levels and coronary plaque inflammation, independently of serum LDL cholesterol levels [44]. Moreover, the PCSK9 levels in acute coronary syndromes (ACS) seem to be modulated by inflammation, lipid-lowering therapy, and the clinical onset of ACS. Indeed, in the acute phase of patients with ACS early high PCSK9 plasma levels are associated with high C-reactive protein levels [45]. Recently, high circulating levels of PCSK9 were found to be associated with increased downstream signaling activation, a mechanism that seems to be related to an ROS-mediated pathway, in patients with atrial fibrillation (AF). In this context, PCSK9 levels can discriminate patients at increased risk of cardiovascular events [46]. In particular, patients with higher PCSK9 levels showed a significantly increased rate of ROS and ox-LDL generation [47]. Moreover, a post-hoc analysis of a prospective, single-centre cohort study of 907 patients with non-valvular atrial fibrillation (AF), demonstrated, in vivo, that circulating levels of PCSK9 and LPS are associated with a mechanism possibly involving NADPH oxidase activation, as both LPS and PCSK9 are correlated with Nox2 activation, and patients with concomitant increase in PCSK9 and LPS showed a higher risk of CVEs [48].

### 2.3. Anti-Oxidant Effect of PCSK9-I

Since recent studies have shown an association between PCSK9 and oxidative stress, PCSK9 inhibition could represent a novel therapy to reduce oxidative-stress-associated cardiovascular risk. In a clinical study, Lankin et al. reported the effect of evolocumab on reducing the plasma concentration of ox-LDL in patients with coronary artery diseases without affecting the activity of antioxidant enzymes, including glutathione peroxidase, superoxide dismutase and catalase, in erythrocytes of patients [49]. Similarly, other authors demonstrated that alirocumab modulates oxidative stress by decreasing the hepatic level of lipid peroxidation products in a rat model of alcohol-induced liver injury [49]. The antioxidant effects of evolocumab during oxidative stress condition were investigated in endothelial cells. It was shown to be able to counteract the damage caused by H_2_O_2_ in human umbilical vein endothelial cells (HUVEC) [50]. In a multicenter before–after study in 80 heterozygous familial hypercholesterolemia (HeFH) patients, after 6 months treatment with PCSK9i, oxidative stress was significantly inhibited [51]. Indeed, both Nox2 activation and ox-LDL production were lower compared with basal value [51].

## 3. PCSK9 and Thrombotic Process

### 3.1. The Role of the Thrombosis in the Atherosclerotic Cardiovascular Disease

The pathogenesis of arterial thrombosis is complex and dynamic and originates in an injured atherosclerotic plaque. Thrombus formation involves the release of prothrombotic molecules (such as tissue factor), platelet adhesion to the vascular wall and platelet aggregation and recruitment. This process, along with the activation of coagulation cascade, which is, responsible for fibrin production in the damaged site, leads to the formation and growth of thrombus. The platelets’ role in this process, namely, hemostasis, depends on their capacity to respond to the specific receptors expressed on damaged endothelium [52]. Collagen-mediated platelet thrombus formation occurs when endothelial cells expose collagen to the circulation [53]. Platelets, through their glycoproteins, interact with collagen and collagen-deposited von Willebrand factor (vWF), shape change and adhere to the site of injury. This event leads to the secretion of alpha-granule and release of ADP, serotonin and TxA_2_, leading to the recruitment of more platelets [54]. Activated platelets release thrombin and clotting activation. Deeper tissue damage leads to the release of Tissue Factor (TF) from smooth muscle and adventitial layer. TF mediates thrombin generation, leading to, in turn, platelet activation, fibrin generation and, finally, the coagulation cascade activation implicated in thrombus formation [53]. Enhanced platelet reactivity and thrombosis risk are also associated with pathophysiological conditions, such as atherosclerosis, hyperlipidemia, and hyperglycemia [55,56]. Risk factors implicated in thrombosis, such as hypertension, diabetes, smoking and, newly, Coronavirus Disease 2019 (COVID-19), can contribute to the growth of thrombus through the amplification and propagation of platelet aggregation [57].

### 3.2. PCSK9 and Platelet Activation

Pre-clinical and clinical data support the hypothesis that circulating PCSK9 could affect the platelet activation pathway using different mechanisms.

PCSK9 knockout mice show reduced carotid artery thrombosis induced by FeCl3, with the formation of non-occlusive thrombi [58] (70% of PCSK9 knockout mice after 30 min, while 57% of Wt mice showed total artery occlusion before 15 min after FeCl3 administration, suggesting an impaired platelet function) [58]. In line with this, PCSK9 enhances in vivo thrombosis in a FeCl3-injured mesenteric arteriole thrombosis mouse model, while PCSK9 inhibitor evolocumab inhibits its enhancing effects [59]. Notably, platelets from PCSK9 knockout mice show a reduced platelet activation, as demonstrated by the significant reduction in glycoprotein IIb/IIIa expression levels, sP-selectin expression levels and circulating platelet-leukocyte aggregates compared to Wt mice [58]. In addition, von Brühl et al. showed that thrombi generated by the ligation of inferior vena cava in PCSK9 knockout mice models have less leukocyte enrolment, a phenomenon dependent upon sP-selectin, which results in downregulated PCSK9 knockout mice [60]. Soluble P-selectin (sP-selectin) and soluble CD40 ligand (sCD40L) are markers of platelet activation associated with cardiovascular risk [61]. In animal model experiments conducted on 30 male rabbits with dyslipidemia, treated with 10-Dehydrogingerdione, a novel cholesteryl ester transfer protein (CETP) inhibitor, which is able to suppress PCSK9 expression, induced a marked decrease in the soluble sCD40L and sP-selectin [62]. Indeed, the reduction in these markers showed a significant correlation with PCSK9 suppression [62]. This is further evidence that PCSK9 is involved in platelet activation. Several human perspective studies investigated the role of PCSK9 in platelet function and the onset of cardiovascular outcomes. Navarese et al. explored platelet aggregation and the onset of major adverse cardiovascular events (MACEs) in PCSK9-REACT patients with ACS receiving prasugrel or ticagrelor, P2Y12 inhibitors, and undergoing percutaneous coronary intervention (PCI) [63]. Their data showed that increased PCSK9 levels are associated with higher platelet reactivity and are a possible predictor of ischemic events in ACS patients undergoing PCI. In more detail, patients with the highest PCSK9 plasma levels had a 2.62-fold risk of developing recurrent coronary events in comparison with those with lower PCSK9 plasma levels [63]. These results were corroborated by experiments in which human recombinant PCSK9, added to healthy human platelet-rich plasma, was able to significantly increase platelet aggregation and reduced aggregation lag time after stimulation with a subthreshold concentration of epinephrine (0.3 and 0.6 mM) [58]. Similarly, platelets incubated with PCSK9, at the concentration found in the circulation of atrial fibrillation (AF) patients, increased platelet aggregation and platelet thromboxane B_2_ (TxB2) release [47], a marker of in vivo platelet activation [64]. 

In patients with stable coronary artery disease (CAD), a relationship was found between PCSK9 plasma levels and total number of circulating platelets [65]. Similarly, a direct relationship between in vivo platelet activation, PCSK9 plasma levels and cardiovascular events incidence has been reported in AF patients [46]. AF patients with PCSK9 above the median levels have a higher rate of platelet aggregation and recruitment, coinciding with higher levels of thromboxane B_2_ expression and P-selectin release [26]. Altogether, these data support the hypothesis that increased PCSK9 levels correlates with impaired platelet reactivity and subsequent higher atherothrombotic risk.

Despite all this observational evidence, whether PCSK9 exerts a direct effect on platelets or the effects result from dyslipidemia generated by PCSK9 binding to LDLR is still debated. It is known that PCSK9 is involved in lipid metabolism and the atherothrombotic process binding LDLR [66], but its pleiotropic effect on cellular mechanisms involved in atherogenesis and plaque complications is still being studied. A direct effect of PCSK9 on platelets, independent of its lipid-lowering effects, was recently demonstrated.

Dyslipidemia induces ox-LDL generation and, in turn, facilitates platelet activation by binding scavenger receptors including CD36 and LOX1 [35,67]. PCSK9-induced platelet activation involves these two important receptors on the platelet surface [35,67]. Platelets’ incubation with PCSK9 increased PA, oxidative stress and p38, p47 and Phospholipase A2 (PLA2) phosphorylation. These changes were amplified by exogenous LDL and blunted by a CD36 inhibitor. Additionally, co-immunoprecipitation analysis revealed an immune complex of PCSK9 with CD36. The same results were confirmed by Qi et al., who, through in vitro and in vivo experiments, showed that PCSK9 directly enhances agonist-induced platelet aggregation, dense-granule ATP release, and integrin αIIbβ3 activation P-selectin release from α-granules [59]. However, a large in vivo study demonstrated the correlation between serum PCSK9 levels and urinary markers of platelet activation, independently of circulating levels of lipids [46].

Mechanism studied revealed that PCSK9 binds platelet CD36, and thus activates Src kinase and mitogen-activated protein kinase (MAPK)—extracellular signals, increasing the generation of ROS and activating the signaling pathways downstream of CD36. The CD36 knockout mice corroborated that the enhancing effects of PCSK9 on platelet activation are CD36-dependent [59]. Hence, PCSK9 in the plasma directly enhances platelet activation and in vivo thrombosis by binding to platelet CD36 and thus activating the downstream signaling pathways.

Evidence has shown the role of PCSK9 in LOX1 signaling modulation in different cell lines. Murine models demonstrated that PCSK9 induces LOX1 expression on primed macrophages, favoring ox-LDL uptake [41]. The same process was observed on human vascular endothelial cells [68]. 

**Figure 1:**
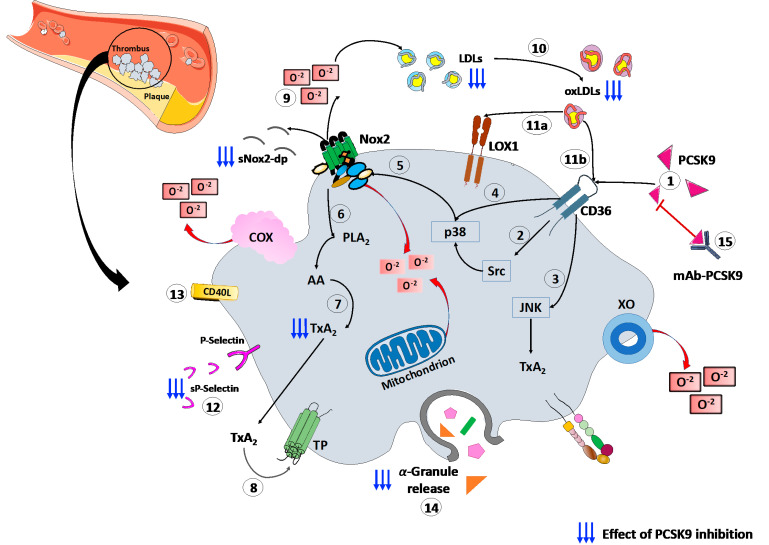
PCSK9 effects on platelet activation. (1) PCSK9 directly binds CD36 receptor on platelet’s surface, enhancing platelet activation and the downstream signaling, including (2) Src and (3) JNK kinase. Moreover, PCSK9 increases the generation of ROS by (4) p38MAPK phosphorylation inducing (5) Nox2 activation, (6) PLA2, (7) AA and (8) TxA2 signaling. (9) The Nox2-mediated ROS production increases (10) ox-LDLs formation that amplify the platelet activation through both (11a) LOX1 and (11b) CD36 platelet receptors. All these events act as the amplifying signal for platelet activation leading to (12) p-selectin expression, (13) CD40L expression and (14) release of granule contents. (15) mAbs-PCSK9 inhibit all these mechanisms. Abbreviations. AA: arachidonic acid; CD40L: CD40 ligand; Gp: glycoprotein; H_2_O_2_: hydrogen peroxide; JNK: c-Jun N-terminal kinase; LDL: low-density lipoproteins; MAPK: mitogen-activated protein kinase; ox-LDLs: oxidized low-density lipoproteins; PCSK9: proprotein convertase subtilisin/kexin 9; PLA2: phospholipase A2; ROS: reactive oxygen species; sNOX2-dp: soluble NOX2-derived peptide; TP: thromboxane receptor; TxA2: thromboxane A2; LOX-1: Lectin-like ox-LDL receptor-1; mAbs: monoclonal antibodies.

Finally, a recent study, conducted on patients with CAD, proved the platelets’ ability to store PCSK9 and release the stored PCSK9 when activated. The released PCSK9 promotes platelet aggregation and thrombus formation, monocyte chemotaxis and monocytes differentiation into macrophages/foam cells [69]. These findings, together, confirm the role of platelets–PCSK9 interaction at different stages of the atherothrombotic process. (Figure 1).

### 3.3. Anti-Platelet Effect of PCSK9-I

PCSK9i effects on platelet activity highlight the interplay between PCSK9 and platelet function modulation in humans. PCSK9i reduces oxidative stress, inhibiting Nox2 activation and blocking acid arachidonic signaling, and inhibits platelet activation in human platelets of healthy subjects [51]. Barale et al. [70] demonstrated that 6 months of PCSK9i administration reduced platelet activation. They found a decrease in circulating levels of platelet activation markers, such as the expression of CD62P, plasma levels of soluble CD40L, Platelet Factor-4, and sP-Selectin, and a correlation between these markers and serum PCSK9 in 23 HeFH patients after PCSK9i treatment [70]. These results were recently confirmed by a multicenter before–after study conducted on a larger population of HeFH patients, where PCSK9i treatment reduced platelet activation, modulating Nox2 activity and, in turn, ox-LDL formation, as proven by the lowered platelet aggregation after treatment and the independent correlation between PCSK9 and serum TXB_2_ decrease [51] (Figure 1).

During the pandemic, thrombotic COVID-19 complications caught researchers’ interest. Patients with HeFH showed a higher risk of developing severe COVID-19, probably due to LDL-receptor variants’ modulation of the long-term immune response to COVID-19 [71]. Despite the hypothesis that statins or PCSK9i, by their lipid-lowering, antioxidative and antithrombotic properties, could protect against COVID-19 complications [71], no data were reported.

The main studies evaluating the role of PCSK9 on platelet function are summarized in Table 1.

## 4. Conclusions

Exciting novel findings suggest that the PCSK9 is an unknown major player in the atherothrombotic process. Although initially found to regulate cholesterol metabolism by LDLR modulation, accumulating evidence demonstrates that PCSK9 is also able to directly and indirectly modulate several pathways involved in ROS production and platelet activation, as represented in Figure 1.

Thus, PCSK9 is deeply involved in the atherothrombotic process, and the introduction of PCSK9i gave us a new tool to counteract this phenomenon, not only by lowering LDL cholesterol but also by inhibiting platelet activation and thrombus formation, as demonstrated by several human, in vitro and animal models.

However, the availability of this new drug class must not let us forget that prevention comes first. Indeed, cardiovascular prevention strategies showed favorable effects on circulating levels of PCSK9. For instance, high adherence to the Med-Diet is associated with lower levels of PCSK9 [48]. In particular, extra-virgin olive oil and wine, two foods rich in antioxidant compounds, showed an independent, inverse association with PCSK9 levels [48] and resveratrol, the main red wine polyphenol, reduces PCSK9 liver expression [72], suggesting supplementation with a Med-diet and/or antioxidants as a helpful tool to regulate PCSK9 level and reduce CV risk.

## Figures and Tables

**Table 1:** Pre-clinical and clinical studies on PCSK9 and platelet function.

References	Study Design	Platelet Actvation Markers	Main Results
***In vitro studies***			
Camera et al. (2018) [58]	PRP of HS +STC of epinephrine (0.3–0.6mM) +hrPCSK9 (5mg/mL)	↑ Aggregation ↓ Lag phase ↑ GPIIb/IIIa ↑ P-selectin ↑ platelet–leukocyte aggregates	PCSK9 induced an increase of platelet reactivity
Cammisotto et al. (2020) [47]	wPLT from HS +PCSK9 (1.0–2.0 ng/mL) alone or with +LDL (50 μg/mL)	↑ Platelet aggregation ↑ TxB2 ↑ cPLA2 Phosphorylation	wPLT from HS +PCSK9 (1.0–2.0 ng/mL) alone or with +LDL (50 μg/mL)
Qi et al. (2021) [59]	(1) Human wPLT +PCSK9 +PLT agonists (ADP, thrombin, collagen)	(1) ↑ Platelet aggregation ↑ Src, ERK, JNK, p38, and cPLA2 phosphorylation ↑ TxB2	(1) Human wPLT +PCSK9 +PLT agonists (ADP, thrombin, collagen)
	(2) WT mouse PLT vs. CD36^−/−^ mouse PLT	(2) ↓ Platelet aggregation ↓ Src, ERK, JNK, p38, and ↓ cPLA2 phosphorylation ↓ TxB2	(2) WT mouse PLT vs CD36^−/−^ mouse PLT
Cammisotto et al. (2021) [51]	wPLTs from HS +plasma from HeFH after PCSK9i	↓ Platelet aggregation ↓ TxB2	PCSK9i treatment reduces platelet activation in HeFH patients.
Petersen-Uribe et al. (2021) [69]	(1) PLTs CRP-stimulated	(1) ↑ CD62P expression ↑ PCSK9 release	Platelets are source of PCSK9 Platelet-derived PCSK9 contributes to atherothrombosis Inhibition of PCSK9 attenuates athero-thrombotic process
	(2) PLT PCSK9i-treated	(2) ↓ Platelet aggregation CRP- induced ↓ Thrombus formation	
	(3) SPN derived to platelets rhPCSK9-stimulation	(3) ↑ Monocytes migration	
	(4) co-culture Platelets/Monocytes rhPCSK9-stimulated	(4) ↑ Macrophages differentiation	
***Animals studies***			
El- Seweidy et al. (2019) [62]	30 Dyslipidemic rabbits 10 Normal rabbits + 10-Dehydrogingerdione	↓ sCD40L ↓ sP-selectin	Reduction of PA markers correlated with PCSK9 levels
Camera et al. (2018) [58]	PCSK9 ^−/−^ mice vs. PCSK9 ^+/+^ mice	↓GPIIb/IIIa ↓P-selectin ↓platelet–leukocyte	Occlusion of carotid artery with non-occlusive thrombi formation
Qi et al. (2021) [59]	(1) WT mice MI mice model +hrPCSK9	(1) ↑platelet aggregation, ↑ATP release ↑integrin αIIbβ3 activation ↑ sP-selectin ↑spreading and clot retraction	PCSK9 enhances platelet activation and in vivo thrombosis
	(2) WT vs. LDLR^−/−^ mice +hrPCSK9	(2) ↑ increased thrombus formation	
***Human studies***			
Li et al. (2015) [65]	330 CAD patients	↑ Platelet cout	Association between plasma PCSK9 levels and PLT count
Pastori et al. (2017) [46]	907 AF patients	↑ 11-dh-TxB2	Correlation between PCSK9 and 11-dh-TxB2 Association between PCSK9 levels and increased risk of CVEs
Navarese et al. (2017) [63]	178 ACS patients with follow-up to 1 year	↑ Aggregation	PCSK9 serum levels were associated with MACEs and platelet reactivity
Barale et al. (2020) [70]	24 HeFH +mAbs anti-PCSK9	↓ sCD40L ↓ sP-selectin ↓ CD62P ↓ PF-4,	Correlation between platelet activation markers and serum PCSK9
Cammisotto et al. (2020) [47]	88 AF patients: 44 AF < 1.2 ng/mL PCSK9 levels 44 AF > 1.2 ng/mL PCSK9 levels	↑ Platelet aggregation ↑ Recruitment ↑ TxB2 ↑ sP-selectin In 44 AF < 1.2 ng/mL PCSK9 levels	Circulating levels of PCSK9 are significantly positively associated with markers of platelet activation
Qi et al. (2021) [59]	Ex vivo study (n = 102) PRP from patients with low PCSK9 level vs. high PCSK9 level	↑ Platelet aggregation	high PCSK9 levels increased platelet aggregation mAbs anti-PCSK9 inhibited this effect
Cammisotto et al. (2021) [51]	80 HeFH Before-after mAbs anti-PCSK9	↑ TxB2 ↑ ox-LDL	Correlation between ox-LDL and PCSK9 levels

Abbreviations: proprotein convertase subtilisin/kexin 9 (PCSK9), soluble CD40 ligand (sCD40L), soluble P-selectin (sP-selectin), low-density lipoprotein cholesterol (LDL-C), high-density lipoprotein cholesterol (HDL-C), platelet-rich plasma (PRP), healthy subjects (HS), glycoprotein (GP), subthreshold concentration (STC), human recombinant PCSK9 (hrPCSK9), acute coronary syndrome (ACS), Major adverse cardiovascular events (MACEs), atrial fibrillation (AF), Thromboxane B2 (TxB2), washed platelet (wPLT), coronary artery disease (CAD), 11-dehydro-thromboxane B_2_ (11-dh-TxB_2_), cardiovascular events (CVEs), monoclonal antibodies (mAbs), Platelet Factor-4 (PF-4), wild type (WT), myocardial infarction (MI), phospholipase A2 (cPLA2), heterozygous familial hypercholesterolemia (HeFH), oxidized-LDL (ox-LDL), PCSK9 inhibitors (PCSK9i), Supernatant (SPN). (↑) Increased; (↓): decreased; (+): added.

## Data Availability

Not applicable.
